# Vitamin D deficiency and the vitamin D receptor (VDR) gene polymorphism rs2228570 (FokI) are associated with an increased susceptibility to hypertension among the Bangladeshi population

**DOI:** 10.1371/journal.pone.0297138

**Published:** 2024-03-14

**Authors:** Imrul Kayes Nabil, Zimam Mahmud, Sonia Tamanna, Md Mostafijur Rahman, Gowhar Rashid, Md. Sarowar Hossain, Humaira Binte Asad, Mohammad Amjad Kamal, Md. Zakir Hossain Howlader

**Affiliations:** 1 Department of Biochemistry and Molecular Biology, University of Dhaka, Dhaka, Bangladesh; 2 Department of Medical Laboratory Technology, Amity Medical School, Amity University Haryana, (AUH), Gurugram, India; 3 Department of Pharmacy, Daffodil International University, Birulia, Bangladesh; 4 Institutes for Systems Genetics, Frontiers Science Center for Disease-Related Molecular Network, West China Hospital, Sichuan University, Chengdu, China; 5 King Fahd Medical Research Center, King Abdulaziz University, Jeddah, Saudi Arabia; 6 Enzymoics, Hebersham, NSW, Australia; 7 Novel Global Community Educational Foundation, Hebersham, New South Wales, Australia; University of Catania: Universita degli Studi di Catania, ITALY

## Abstract

Vitamin D receptor (VDR) gene is implicated in hypertension vulnerability due to its role in regulating the renin-angiotensin system (RAS) and blood pressure. In this case-control study, a carefully selected cohort of 111 hypertensive individuals and 100 healthy controls underwent serum analysis using HPLC to measure 25-hydroxy vitamin D levels. Polymorphic variations in the VDR gene were detected and characterized using the PCR-RFLP method. At first, lower 25-hydroxy vitamin D levels were observed in hypertensive individuals compared to controls (p<0.001). The genotype frequency of the VDR gene TaqI showed no significant difference between cases and controls (p>0.05). Similarly, no significant difference was found in the VDR gene BsmI genotype frequency between hypertensive patients and controls (p>0.05). However, a statistically significant distinction was observed in the VDR gene FokI genotype frequency between cases and controls (p<0.01). The odds ratios for FokI genotypes (CC, CT, TT, and CT+TT) were 1.0, 0.590, 1.566, and 0.963, respectively. Furthermore, serum 25-hydroxy vitamin D levels were significantly higher in control subjects compared to hypertensive patients across all genotypes of VDR (p<0.001). Hypertensive patients, excluding those with the FokI VDR gene CC genotype, exhibited significantly higher systolic blood pressure levels compared to the control group (p<0.05). Similarly, hypertensive subjects displayed elevated diastolic blood pressure levels compared to the control group (p<0.001). Overall, the results suggest the presence of a potential inverse correlation between serum 25-hydroxy vitamin D levels and hypertension. The association analysis conducted indicated that there is no significant association between TaqI and bsmI genotypic variants and the risk of developing hypertension. However, it was observed that VDR gene polymorphisms do have a clear association with hypertension susceptibility, as evidenced by the significantly higher occurrence of FokI genotypic variants in hypertensive patients. Our study therefore introduces the possibility of utilizing 25-hydroxy vitamin D deficiency and VDR gene polymorphisms as a biomarker for hypertension.

## Introduction

Hypertension is one of the foremost public health concern in both developed and developing countries around the world. In general, a transient or chronic elevation of blood pressure in the arteries that may cause cardiovascular damage is referred to as hypertension. Long-term hypertension may lead to heart failure, myocardial ischemia, stroke, metabolic syndrome, aneurysm, chronic renal disease, peripheral arterial disease, vision loss and other symptoms [1, 2]. Genetic variation, obesity, family history, environmental factors have been identified as the major risk factors of hypertension. Furthermore, regulation of renin-angiotensin-aldosterone system (RAAS) by vitamin D has been previously demonstrated [3] and thus, downregulated RAAS activation is a key contributor to the development of hypertension. According to a recently published report, approximately 40.7% of adult people suffer from hypertension in Bangladesh [4]. To date, vitamin D deficiency is abundant in the Bangladeshi population, and preceding study has revealed that low vitamin D levels augment the risk of hypertension [5].

Vitamin D is produced from its precursor, cholesterol, where 7-dehydrocholesterol (an intermediate in the pathway of cholesterol synthesis) is converted to pre-vitamin D3 by UV irradiation [6]. It is converted slowly and non-enzymatically to cholecalciferol (vitamin D3). The cholecalciferol then carried in the bloodstream to liver where it is converted to calcidiol which is further converted to calcitriol in the kidney, the biologically functional form of vitamin D. Additionally, 25-hydroxy vitamin D binding protein (DBP), a plasma protein in the circulatory system, binds to calcitriol and transports it to various target organs [6, 7].

Notably, the vitamin D receptor (VDR), a ligand-induced transcription factor belonging to the nuclear receptors family, is involved in a multitude of pathological process and is primarily responsible for vitamin D function [8, 9]. The VDR usually binds to its ligand 1,25(OH)_2_D in particular and cooperates with specific nucleotide sequences of different target genes to induce a plethora of biochemical functions. The VDR receptor gene, which has at least five promoter regions and at least 11 exons that cover kb of DNA, is found on the long arm of chromosome 12 (12q13.11) [10]. Of all 11 exon sequences, while exons 2–8 encode the VDR protein, the first exon is not translated. Most importantly and in relevance to our study, the VDR gene has been identified to have more than 470 genetic mutations or single nucleotide polymorphisms (SNPs) till date [11]. The most significant and extensively studied SNPs of VDR are, FokI, rs2228570; BsmI, rs1544410; TaqI, rs731236; ApaI, rs7975232; and Poly(A) rs17878969 [11].

The polymorphic site of FokI restriction enzyme is located on exon 2 in the 5’ coding region of the VDR gene [12], and the genotypes were identified as CC, CT and TT for the FokI polymorphisms. In addition, the mutational landscape of BsmI restriction enzyme is located also in exon 2 of the VDR gene, however, it does not affect the quality of the translated VDR protein [13]. Similar to FokI, the genotypes assigned are CC, CT and TT for the BsmI polymorphisms. In the TaqI polymorphism, a Thymine at codon 352 in exon 9 of the VDR gene is converted into Cytosine nucleotide (ATT to ATC) [14]. Genotypes of TaqI polymorphisms are generally designated as TT, TC and CC [15]. It is noteworthy that some SNPs in VDR may serve as a genetic predisposition in causing several number of diseases. Mutation in the VDR gene may modify receptor length and suppress its activation in target cells [16].

In Bangladesh, a significant number of individuals are experiencing hypertension, and the incidence of this condition is steadily rising. Furthermore, no previous research has been conducted in the Bangladeshi population to investigate the potential correlation between hypertension and 25-hydroxy vitamin D and its receptor VDR. Therefore, our study not only examined the frequency of VDR gene polymorphisms in hypertensive patients but also explored the impact of these polymorphisms on serum 25(OH)D levels, blood sugar levels, and blood pressure patterns. The outcomes of this study have the potential to enhance the treatment approach for hypertension, leading to early detection, improved management, and better control of the condition.

## Materials and methods

### Selection of study population

The present research was conducted with two study population e.g. hypertensive patients and healthy people with normal blood pressure. A total number of 111 hypertensive patients and 100 healthy people, all having a Bengali ethnic background, were registered in this study. The sample size was calculated using Cochran’s formula according to Daniel and Naing et al. [17, 18]. The calculated sample size using the above formula was 225 for each group. However, we couldn’t collect all the samples due to the short period of time allocated for the study. The case samples were collected from patients of Bangabandhu Sheikh Mujib Medical University Hospital (BSMMUH) and Dhaka Medical College Hospital (DMCH) in Bangladesh between September 2018 and March 2019. There was no inherent risk associated with this study as we conducted it by sampling diagnosed cases, which is a standard protocol followed at both of these hospitals. The control samples were collected from the healthy individuals of the Department of Biochemistry and Molecular Biology, University of Dhaka, and from the two hospitals of Dhaka city (BSMMUH and DMCH) while they came for a regular checkup between September 2018 and March 2019. Written consent was taken from all the study subjects. Data collection involved administering a structured questionnaire through face-to-face interviews conducted by the researcher. The questionnaire encompassed socio-demographic aspects such as age, presence of any complications, blood pressure, weight, height, BMI, and other relevant information. The study subjects were also well informed that only the research project members had access to information that could identify individual participants during or after data collection. The study was granted approval by the Institutional Ethical Review Committee of the Department of Biochemistry and Molecular Biology at the University of Dhaka (BMBDU-ERC/EC/18/020). The study adhered to the principles outlined in the declaration of Helsinki and its subsequent revisions [19].

### Sample collection and storage

Blood samples from hypertensive patients and control individuals were taken from each participant. A 5 mL disposable syringe was used to draw about 5 mL of venous blood while taking all necessary aseptic measures. The collected blood was allowed to stand at room temperature for a few minutes in the test tube (the duration between sample collection and processing is ≤ 2 hours). The plain tube was then centrifuged for 10 minutes at 3,000 rpm. Serum was then transferred with a micropipette taking care of not taking any red cell. Appropriate aliquots of serum were stored in Eppendorf tubes. All samples (serum and whole blood) was stored at -20°C.

### Determination of total 25-hydroxy vitamin D level in serum by HPLC

At first, 0.5 mL of serum was taken into a screw-capped tube. Then, 350 μL of methanol: 2-propanol (80:20) were added to 0.5 mL of serum solution. A Multitude vortex mixer (Digisystem; VH-200) was used to combine the mixture of tubes for 30 seconds. The 25(OH)D was extracted by mixing it with 2 mL of hexane three times for 60 seconds each. Centrifugation (Digisystem) was used to separate the phases, and the top organic phase was moved to a conical tube and dried in liquid nitrogen. 100 μL of methanol was used to dissolve the residue. Finally, total level of 25(OH)D was determined in HPLC using the protocol discussed previously [20]. A UV detector was then used to detect 25(OH)D at 265 nm.

### DNA extraction and quantification

DNA was extracted from blood samples according to the procedure discussed previously [21]. Absorbance of diluted DNA was measured at 260 nm and 280 nm. The consistency of DNA of the samples was also evaluated by agarose gel electrophoresis. A gel containing 0.5% agarose was used for this purpose. The electrophoresis was performed at low voltage (40 V) for an hour and visualized using CLEAVER Gel Documentation System. After that the DNA was stored in -20°C for further use.

### Genotyping of VDR gene polymorphisms

At first, polymerase chain reaction (PCR) was performed for the purpose of genotyping. To determine the mutational landscapes as well as VDR genotypes the restriction fragment length polymorphism (RFLP) was used. Amplification of genomic DNA was performed by PCR using the following primers which can create DNA fragment containing expected cutting sites. Primer sequences are mentioned in Table 1.

**Table 1:** List of primer sequences used in the study.

*Locus*	*Forward Primer*	*Reverse Primer*
*TaqI*	5’-CAACCAAGACTACAAGTACCGCGTCAGTGA-3’	5’-CACTTCGAGCACAAGGGGCGTTAGC-3’
*BsmI*	5’-AGTGTGCAGGCGATTCGTAG-3’	5’-ATAGGCAGAACCATCTCTCAG-3’
*FokI*	5′-AGCTGGCCCTGGCACTGACTATGCTCT-3′	5′-ATGGAAACACCTTGCTTCTTCTCCCTC-3′

PCR was performed according to the conditions described previously [22]. After completion of PCR, 3 μL of the PCR product was tested for amplification on a 2% agarose gel in a Gel-Doc machine (Life Technologies). By comparing the product’s size to a 100 bp DNA ladder (Thermo Scientific, USA), the ideal size was determined. The gel was stained with ethidium bromide solution, and the amplified DNA was visualized under a UV Transilluminator while the gel image was captured.

### Restriction digestion of PCR product

VDR gene candidate polymorphic markers at TaqI rs731236 (T>C), BsmI rs1544410 (C>T) and FokI rs2228570 (C>T) were analyzed using site-directed restriction enzymes TaqI, BsmI and FokI respectively. Restriction digestions was carried out according to the methods described earlier [23]. Following the typical digestion protocol, the genotypic variants of VDR were analysed and determined by RFLP assay. In brief, the digestion took place in a reaction volume of 15 μL. The water bath was adjusted at 65°C for both TaqI and BsmI while for FokI digestion was performed at 37°C. The digestion tube with reaction volume was incubated for 16 hours for the completion of full digestion. After ethidium bromide staining, the product of enzyme digestion was resolved in agarose gel (2%) and visualized using gel documentation system.

### Statistical analysis of the data

Experimental data were stated as mean ± SEM (Standard Error of Means). Data analyses were carried out using the Graphpad Prism (version 8.0) software. Using logistic regression models, odds ratios (OR), a measure of relative risk, were estimated at 95% confidence intervals (CI). The statistical method used was student’s t-test (two-tailed), Chi-square test, Fisher’s exact test and one-way ANOVA. Differences were considered significant when p<0.05.

## Results

### Demographic, anthropometric and clinical data of the study subjects

The demographic, anthropometric and clinical data have been presented in Table 2. The mean age was 39.48 ± 1.235 and 37.80 ± 1.785 (years) in hypertensive patients and control respectively. The mean systolic blood pressure (SBP) was 141.2 ± 16.0 and 109.6 ± 7.09 (mm of Hg) in hypertensive patient and control group respectively. SBP values of hypertensive patients was significantly upper rather than control subjects (p <0.001). Mean diastolic blood pressure (DBP) was 90.5 ± 9.72 and 71.07 ± 6.71 (mm of Hg) in hypertensive patient group and control group respectively. DBP values of hypertensive individuals was significantly increased rather than control subjects (p <0.001). The mean BMI was 26.03 ± 3.79 and 25.95 ± 3.01 (Kg/m2) in hypertensive patients and control group respectively. The mean Random Blood Sugar (RBS) level was 5.28 ± 0.99 and 6.19 ± 1.65 (mmol/L) in hypertensive patients and control group respectively. RBS level of hypertensive people was significantly reduced rather than control subjects (p <0.05).

**Table 2:** Demographic, anthropometric and clinical data of the study subjects.

Parameter	Hypertensive Patients	Control	P Value
**Male (Female)%**	70.42 (29.58)	68.47 (31.53)	-
**Age**	39.48 ± 1.24	37.80 ± 1.79	ns
**BMI (kg/m** ^**2**^ **)**	26.03 ± 3.79	25.95 ± 3.01	ns
**SBP (mmHg)**	141.2 ± 16.0	109.6 ± 7.09	< 0.001
**DBP (mmHg)**	90.5 ± 9.72	71.07 ± 6.71	< 0.001
**RBS (mmol/L)**	5.28 ± 0.99	6.19 ± 1.65	< 0.001
**Serum Total Protein (gm/L)**	53.56 ± 12.63	57.58 ± 5.78	< 0.001
**Serum Calcium (mmol/L)**	2.17 ± 0.11	2.52 ± 0.04	< 0.001

BMI, Body Mass Index; SBP, Systolic Blood Pressure; DBP, Diastolic Blood Pressure; RBS, Random Blood Sugar. ns, non-significant; p <0.05 was considered as level of significance. Result is represented as mean ± SEM.

### Status of biochemical parameters in different study groups

**Fig 1:**
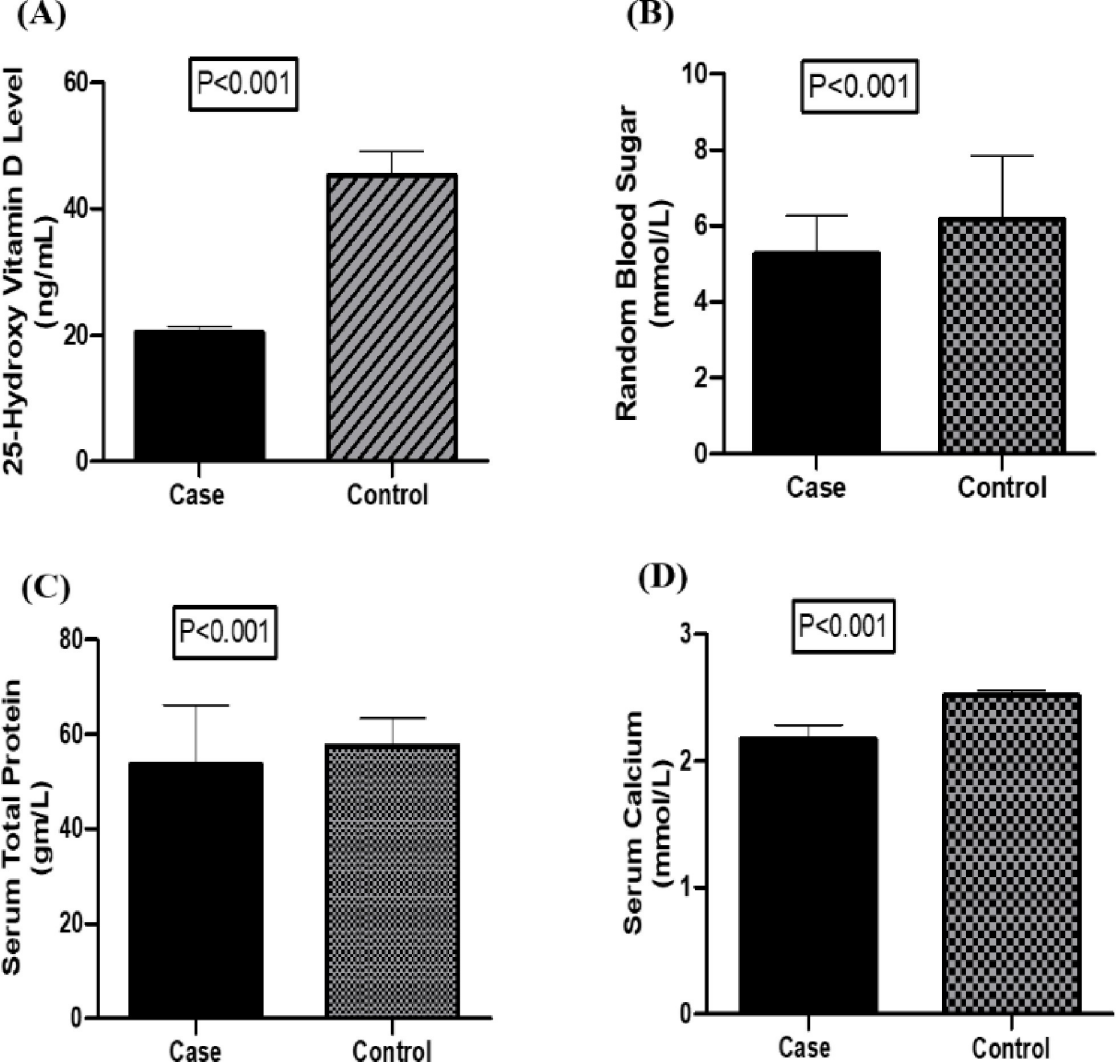
Mean 25-hydroxy vitamin D status, RBS level, Serum total protein and serum calcium level in different study subjects (A-D). Data are presented as Mean ± SEM (standard error of mean). Student’s t-test was performed to analyse data. P<0.05 was considered as level of significance.

Mean 25-hydroxy vitamin D level, random blood sugar level, serum total protein level, calcium level was analysed between cases and controls of our study groups. According to the graphs presented in the Fig 1A, the mean serum 25-hydroxy vitamin D level was 20.36 ± 1.064 and 45.26 ± 3.801 (ng/mL) in hypertensive patient and control subjects respectively. The ranges of 25-hydroxy vitamin D level were (18.15 to 22.22) and (37.45 to 50.75) in hypertensive patient and control subjects respectively. When compared to the control, the 25-hydroxy vitamin D level in the hypertensive people was significantly lower (p <0.001).

Random blood sugar level of individuals with hypertension and control subject was shown in Fig 1B. The mean Random blood sugar level was 5.28 ± 0.99 and 6.19 ± 1.65 (mmol/L) in hypertensive individuals and control subjects, respectively. The Random blood sugar level of the patient group was significantly lesser (p <0.05) compared to the control group. The level of serum total protein and serum calcium levels were found to be significantly lower in hypertensive patients when compared with control groups (p <0.001) (Fig 1C and 1D).

### Correlation and regression analysis between different biochemical parameters

**Fig 2:**
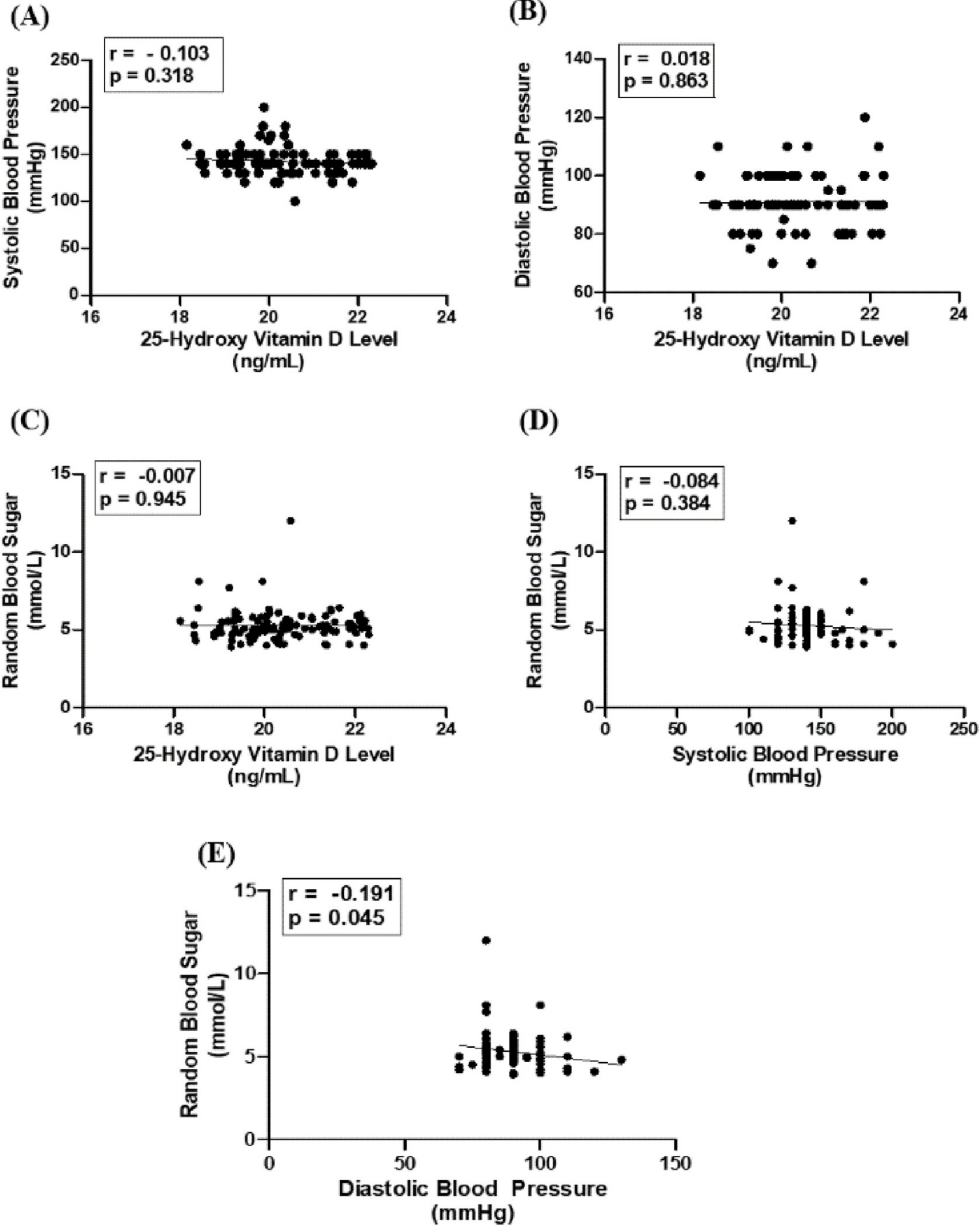
Correlation and regression analysis of 25-hydroxy vitamin D status with SBP, DBP and RBS (A-E). A significant negative correlation was found between RBS and DBP (p <0.05) (Fig 2D).

The Fig 2 shows the association between systolic and diastolic blood pressure and 25-hydroxy vitamin D levels and random blood glucose and 25-hydroxy vitamin D levels as well as the association between random blood sugar and systolic and diastolic blood pressure in the hypertensive population. No significant association was found between 25-hydroxy vitamin D levels and systolic and diastolic blood pressure and random blood glucose in the hypertensive population (r = -0.103, p = 0.318, r = 0.018, p = 0.863 and r = -0.007, p = 0.945 respectively) (Fig 2A–2C). Moreover, there was no significant association between Random blood glucose and Systolic Blood Pressure (SBP) in the hypertensive population (r = -0.084, p = 0.384) (Fig 2D).

Additionally, the correlation coefficient between Random Blood Glucose and Diastolic Blood Pressure (DBP) was also measured as r = -0.1909 in hypertensive group. Apart from others, this result suggested a significant negative correlation (p <0.05) between these two variables in hypertensive patient. Sequential regression analysis also supported the findings showing significant association between these two variables (p <0.05) (Fig 2E).

### Genotypic and allelic frequency distribution of TaqI (rs731236) polymorphisms in hypertensive patients and healthy controls

The genotype frequency of TaqI genotypes and their association with the risk of developing hypertension have been presented in Table 3. Representative picture of agarose gel electrophoresis performed by using PCR products of TaqI digestion are shown in supplementary figure SF1 and SF6 in S1 and S3 Files. In the Table 3, Chisquare test showed no significant difference of genotype frequency (TT, TC and CC) between cases and controls (p> 0.05). No substantial variation was observed between two groups in case of TC, CC and TC+CC genotypes. From the logistic regression model, in individuals having TC genotype, the risk of developing hypertension was less likely compared to their ancestral genotype TT (rs731236 TC: OR = 0.7641, 95% CI = 0.0481–1.415, p = 0.4265, Table 3). In the case of the CC genotype, the risk of developing hypertension was 1.95 when compared to the TT genotype; however, it was not statistically significant (rs731236 CC: OR = 1.945, 95% CI = 0.6590–5.193, p = 0.3077, Table 3). For the dominant model, TC+CC genotype carriers are less likely to develop hypertension when compared to their ancestral TT genotype (rs731236 TC and CC: OR = 0.9118, 95% CI = 0.4910–1.669, p = 0.8787, Table 3). In short, it should be noted that no correlation was observed between the hypertensive group and the control group regarding different genotypes (Table 3). Moreover, genotypic distribution of VDR gene TaqI based on gender was represented in supplementary table ST1 and ST2 in S2 File. Here, Fisher’s exact test was used to analyze significant difference between two study subjects (hypertensive patients and controls). There was no substantial variation among genotypes of VDR gene TaqI in male or female individually (p>0.05).

**Table 3:** Genotype frequency of VDR gene TaqI, BsmI and FokI polymorphisms and estimated risk with hypertension.

Genotypes	Control N (%)	Case N (%)	P value^a^	P value^b^	OR (95% CI)
**VDR gene TaqI**					
**TT**	44 (44.0)	51 (45.95)	0.2209 (ns)	-	1 (Ref.)
**TC**	49 (49.0)	44 (39.64)		0.4265 (ns)	0.7641 (0.4081–1.415)
**CC**	7 (7.0)	16 (14.41)		0.3077 (ns)	1.945 (0.6590–5.193)
**TC+CC**	56 (56.0)	60 (54.05)		0.8787 (ns)	0.9118 (0.4910–1.669)
**VDR gene BsmI**					
**CC**	10 (10.0)	11 (9.91)	>0.9999 (ns)	-	1 (Ref.)
**CT**	58 (58.0)	64 (57.66)		>0.9999 (ns)	0.9933 (0.3822–2.891)
**TT**	32 (32.0)	36 (32.43)		>0.9999 (ns)	0.9960 (0.3638–2.805)
**CT+TT**	90 (90.0)	100 (90.09)		>0.9999 (ns)	0.9943 (0.3851–2.732)
**VDR gene FokI**					
**CC**	9 (9.0)	11 (9.65)	0.0091 (P <0.01)	-	1 (Ref.)
**CT**	56 (56.0)	39 (34.21)		0.4356 (ns)	0.5909 (0.2273–1.596)
**TT**	35 (35.0)	64 (56.14)		0.4117 (ns)	1.566 (0.5935–4.145)
**CT+TT**	91 (91.0)	103 (90.35)		>0.9999 (ns)	0.9639 (0.3735–2.636)

Data represented as number (percentage), N (%). Chi-square and fisher’s exact test were done to assess significant difference between genotypic group and association of genotype with disease condition. OR (95% CI); Odds Ratio (95% Confidence Interval). P value^a^ for chi-square test; P value^b^ for fisher’s exact test. TC+CC = TaqI dominant model; CT+TT = BsmI dominant model; CT+TT = FokI dominant model.

### Genotypic and allelic frequency distribution of BsmI (rs1544410) polymorphisms in hypertensive patients and healthy controls

The genotype frequency and risk of developing hypertension based on rs1544410 genotypes are shown in Table 3. Representative picture of agarose gel electrophoresis performed by using PCR products of BsmI digestion are shown in supplementary figure SF2 and SF7 in S1 and S3 Files. Here Chi-square test showed no significant difference of genotype frequency (CC, CT and TT) between cases and controls (p> 0.05). With regard to the genotypes CT, TT, and CT+TT, there was no discernible variation among the two groups. From the logistic regression model, in individuals having CT genotype, the risk of developing hypertension was less likely compared to their ancestral genotype CC (rs1544410 CT: OR = 0.9933, 95% CI = 0.3822–2.891, p>0.999, Table 3). Similarly, in case of TT genotype, the risk of developing hypertension was 0.99 when compared to the CC genotype, however, it was not statistically significant (rs1544410 TT: OR = 0.9960, 95% CI = 0.3638–2.805, p>0.999, Table 3). For dominant model, CT+TT genotype carriers are less likely to develop to develop hypertension when compared to CC genotype (rs1544410 CT and TT: OR = 0.9943, 95% CI = 0.3851–2.732, p = p>0.999, Table 3). Furthermore, genotypic distribution of VDR gene BsmI based on gender was represented in supplementary table ST3 and ST4 in S2 File. Here, Fisher’s exact test was used to analyze any discernible difference between two study subjects (hypertensive patients and controls). There was no significant difference between genotypes of VDR gene BsmI in male or female individually (p>0.05).

### Genotypic and allelic frequency distribution of FokI (rs2228570) polymorphisms in hypertensive patients and healthy controls

**Fig 3:**
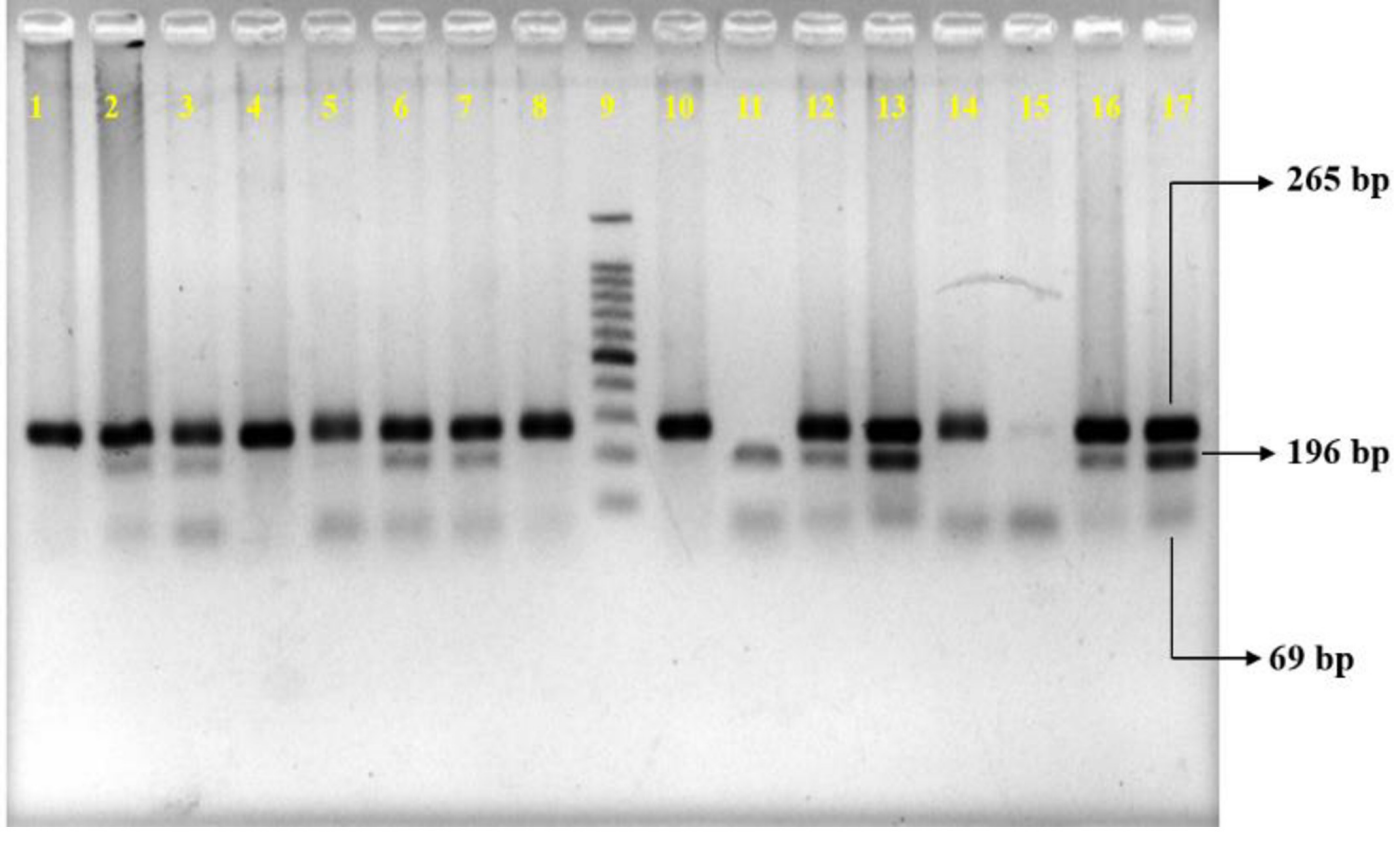
Agarose gel electrophoresis of amplified PCR products upon FokI digestion. Mutation at restriction site 2228570 causes C to T transformation yielding no restriction site. Homozygous wild type (CC) having one restriction site at both alleles created only 196 and 69 bp bands. Heterozygous Ht mutant (CT) created 3 bands (265, 196 and 69 bp.) indicating a restriction site at one allele. Hz mutant (TT) created 265 bp fragment indicating no restriction site at both alleles. A 100 bp DNA ladder was used to compare the size of DNA bands as shown in Lane 9. Digested product was resolved in 2% agarose gel. after ethidium bromide staining. Picture was taken using a UV transilluminator.

The genotype frequency and risk of developing hypertension based on rs2228570 genotypes are shown in Table 3. Representative picture of agarose gel electrophoresis performed by using PCR products of FokI digestion are shown in Fig 3 and supplementary figure SF5 in S3 File. Here, Chi-square test showed a significant difference in genotypic distribution (CC, CT, and TT) between cases and controls (p<0.01). From the logistic regression model, there was no correlation observed in CC, CT, or TT genotypes between the two study groups (OR = 0.5909, 95%CI = 0.2273–1.596 and OR = 1.566, 95%CI = 0.5935–4.145 respectively for CT and TT genotypes compared to CC. p>0.05). Also, the dominant model (CT+TT) showed no significance (OR = 0.9639, 95%CI = 0.3735–2.636, p>0.05). In addition, genotypic distribution of VDR gene FokI based on gender was represented in supplementary tables ST5 and ST6 in S2 File. Here Fisher’s exact test was applied to analyze any discernible difference between two study subjects (hypertensive patients and controls). There was significant difference between genotypes of VDR gene FokI in male (P<0.05) but no significant variations in case of female individually (p>0.05).

### Genotypic distribution and analysis of systolic blood pressure and diastolic blood pressure among study subjects

Analysis of systolic blood pressure level (mmHg) and diastolic blood pressure (DBP) and between control and patient according to genotype are shown in supplementary tables ST7 and ST8 in S2 File. For all three VDR TaqI and BsmI genotypes, systolic blood pressure level was substantially elevated in hypertensive people rather than control subjects (p <0.001). On the other hand, for two VDR FokI genotype (CT and TT), SBP level in hypertensive people was significantly upper rather than control subject (p <0.001). For CC genotype there was no discernible variation among the two-study groups (p >0.05). Similar results were obtained for DBP in individuals with three VDR genotypes.

The mean Systolic Blood Pressure (SBP) level for three genotypes of VDR gene TaqI (TT, TC and CC) was 134.8 ± 3.596, 145 ± 4.382 and 153.3 ± 9.888 mmHg respectively. One-way ANOVA analysis revealed no significant difference among these three genotypic group of TaqI, BsmI and FokI polymorphism among SBP level (p >0.05). Besides, the mean Diastolic Blood Pressure (DBP) level for three genotypes of VDR gene TaqI (TT, TC and CC) was 87.86 ± 2.248, 96.04 ± 2.292 and 98.33 ± 4.773 mmHg respectively. One-way ANOVA analysis revealed the existence of significant difference among these three genotypic group of TaqI polymorphisms among DBP level (p <0.05). On the contrary, no substantial variation was observed among this three genotypic group of BsmI and FokI polymorphism in DBP level (p >0.05).

### Genotypic distribution and analysis of 25-hydroxy vitamin D level among study subjects

Analysis of 25(OH)D level (ng/mL) between control and patients according to genotype is given in Table 4. For hypertensive patient group, mean 25(OH)D level for TaqI TT, TC and CC genotype was 20.33 ± 0.1514, 20.31 ± 0.1511 and 20.56 ± 0.3031 (ng/mL) respectively. For control subject, mean 25(OH)D level for TaqI TT, TC and CC genotype was 44.96 ± 0.6690, 45.43 ± 0.6072 and 45.84 ± 1.391 (ng/mL) respectively. For all three VDR gene, TaqI genotype, 25(OH)D level in the patients with hypertension was significantly reduced rather than control subjects (p <0.001). Similar results were observed in both BsmI and FokI genotypes (p <0.001).

**Table 4:** Genotypic distribution of 25-hydroxy vitamin D level in control and hypertensive patient.

Genotypes	25-hydroxy vitamin D Level (ng/mL)		P-value
	Case	Control	
**TaqI VDR Gene**			
**TT**	20.33 ± 0.1514	44.96 ± 0.6690	P <0.001
**TC**	20.31 ± 0.1511	45.43 ± 0.6072	P <0.001
**CC**	20.56 ± 0.3031	45.84 ± 1.391	P <0.001
**BsmI VDR Gene**			
**CC**	19.94 ± 0.3258	45.82 ± 1.037	P <0.001
**CT**	20.43 ± 0.1341	44.70 ± 0.6205	P <0.001
**TT**	20.35 ± 0.1737	46.00 ± 0.6629	P <0.001
**FokI VDR Gene**			
**CC**	20.23 ± 0.2801	46.73 ± 1.159	P <0.001
**CT**	20.23 ± 0.1770	45.09 ± 0.5727	P <0.001
**TT**	20.46 ± 0.1362	45.04 ± 0.7580	P <0.001

Data represented as mean ± SEM. Unpaired t-test was used to determine significant difference between the two groups. p <0.05 taken as level of significance.

The mean 25(OH)D level for three genotypes of VDR gene TaqI (TT, TC and CC) was 20.33 ± 0.1514, 20.31 ± 0.1511 and 20.56 ± 0.3031 mmHg respectively. One-way ANOVA analysis was applied to see whether any significant difference in 25(OH)D level among these three genotypes was present or not. According to the supplementary figure SF4 in S1 File, we found no significant difference among these three genotypic group among 25(OH)D level (p >0.05) (S1 File). Similarly, no substantial variation was observed among three genotypic group pf BsmI and FokI in 25(OH)D level (p >0.05) (Supplementary figure SF4 in S1 File).

### Haplotype analysis of rs731236, rs1544410 and rs2228570 in study subjects

Table 5 represents haplotype analysis of rs731236, rs1544410 and rs2228570. For VDR gene rs731236 and rs1544410, no haplotypes showed significant association with the risk of hypertension. For VDR gene rs731236 and rs2228570, TC haplotype have protective role in the risk of developing hypertension in patients (P<0.05, OR = 0.572, 95% CI = 0.361–0.907). In Case of VDR gene VDR gene rs1544410 and rs2228570, TT haplotype showed significantly 1.6 times increased risk of hypertension compared to controls (P<0.05, OR = 1.627, 95% CI = 1.053–2.515).

**Table 5:** Haplotype analysis of rs731236, rs1544410 and rs2228570 in study subjects using SheSis web tool.

Haplotypes	Case(freq)	Control(freq)	Chi^2^ Value	Fisher’s p Value	Pearson’s p Value	OR [95% CI]
**VDR gene rs731236, rs1544410**						
**TT**	78(0.351)	50(0.312)	0.629	0.443	0.427	1.191 [0.772–1.837]
**CT**	58(0.261)	44(0.275)	0.089	0.814	0.764	0.932 [0.589–1.474]
**TC**	68(0.306)	58(0.362)	1.328	0.27	0.249	0.776 [0.504–1.194]
**CC**	18(0.081)	8(0.05)	1.416	0.304	0.234	1.676 [0.71–3.957]
**VDR gene rs731236, rs2228570**						
**TT**	98(0.441)	56(0.35)	3.231	0.073	0.072	1.467 [0.965–2.232]
**CT**	63(0.283)	41(0.256)	0.355	0.562	0.55	1.15 [0.726–1.82]
**TC**	48(0.216)	52(0.325)	5.693	0.018	0.017	0.572 [0.361–0.907]
**CC**	13(0.058)	11(0.068)	0.164	0.676	0.685	0.842 [0.367–1.932]
**VDR gene rs1544410, rs2228570**						
**TT**	88(0.396)	46(0.287)	4.841	0.03	0.027	1.627 [1.053–2.515]
**TC**	48(0.216)	48(0.3)	3.469	0.072	0.062	0.643 [0.404–1.024]
**CT**	73(0.328)	51(0.318)	0.043	0.911	0.835	1.047 [0.677–1.617]
**CC**	13(0.058)	15(0.093)	1.695	0.233	0.192	0.601 [0.277–1.301]

Odds Ratio, OR; 95% Confidence Interval, 95% CI; chi-square test, Chi^2^.

## Discussion

The study aimed at identifying the association of VDR gene variants with the risk of hypertension in Bangladeshi individuals. Herein, both patients and controls belonged to the same ethnic background where we have estimated the frequency of VDR genotypes. The levels of serum 25-hydroxy vitamin D, SBP and DBP was also measured to examine their potential involvement as biomarkers of the hypertensive patient in Bangladesh.

Firstly, the baseline characteristics was analyzed between control group and hypertensive patient group (Table 2). Age, sex, BMI, random blood sugar (RBS), systolic and diastolic blood pressure (SBP and DBP), serum total protein, and serum calcium level were the main baseline characteristics of our study. There were 70.4% male and 29.6% female patients in hypertensive patient subject. In control group, 68.5% was male and 31.5% was female. SBPs were found to be 141.2 ± 16.0 mmHg in hypertensive patient and 109.6 ± 7.09 mmHg in control patient group. For DBP, 90.5 ± 9.72 mmHg in hypertensive patient and 71.07 ± 6.71 mmHg in control patient group were observed. Expectedly, there were substantial variations in SBP and DBP between hypertensive and control group (p <0.001). No discernible differences were observed in BMI level of both groups, however, RBS level in hypertensive patient group was significantly lower compared to control group (p <0.05) (Table 2). Sequentially, we found out that serum 25-hydroxy vitamin D level for hypertensive patients (20.36 ± 1.06 ng/mL) are distinctly lower rather than control group (45.26 ± 3.801 ng/mL) (p <0.001) which is correlated with previous findings [24–26]. These data suggest that lack of 25-hydroxy vitamin D in the blood of individuals may act as a risk factor for hypertension in Bangladeshi population.

25-hydroxy vitamin D regulates genes related with glucose metabolism. So adequate level of 25(OH)D is important for proper metabolism of blood sugar. Previous studies showed that 25(OH)D deficiency is linked with the down regulation of glucose metabolism and impaired glucose homeostasis causing type 2 diabetes [27]. However, our study did not find any significant correlation between 25(OH)D level and random blood sugar in hypertensive patients (Fig 2C).

Single nucleotide polymorphisms (SNPs) have been described as one of the important genetic polymorphisms in the VDR gene. Evidences from several clinical and experimental studies support the association of VDR gene polymorphisms with the risk of hypertension. For example, population genetics study suggested that FF genotype and allele F of VDR-Fok I gene are at a greater risk for developing hypertension in Hyderabad, India [28]. Genome-wide association study (GWAS) on VDR gene has proved that FokI (rs2228570) polymorphism is allied with the risk of cardiovascular diseases in the people of European origin (France) which was also validated by *in silico* analyses [29]. Moreover, in a study of Spanish people with hypertension, systolic blood pressure (SBP) with BsmI (rs1544410) CC genotype was found to be higher than TC or TT genotypes in men but not in women [30]. Another very recent study performed by Paula González Rojo et al. revealed a significant association of FokI polymorphism in the people with cardiovascular diseases (CVD) from Southern Spain [31]. In the Korean population, patients with BsmI T allele had higher SBP and DBP, and a concomitant higher risk of hypertension than patients with CC genotypic variants. Another contemporary studies revealed the linkage of BsmI and FokI polymorphisms with hypertension in American men [32]. Importantly, the study showed an inverse linkage of VDR genetic polymorphisms with the susceptibility to hypertension [32]. On the contrary to above data, studies on Chinese Han population suggests that FokI polymorphism in the VDR gene is allied with reduced risk of hypertension [33]. Overall, all these data suggest the involvement of VDR gene polymorphisms in hypertension.

Keeping the above-mentioned literatures in mind, at first we studied the genetic predisposition of VDR TaqI genotypes in hypertension. Among the three TaqI genotypes (TT, TC and CC), the CC genotype was found to have higher frequency in hypertensive patients rather than control subjects but the difference was not significant (p>0.05) (Table 3). In support of this, enhanced frequency of CC genotype was identified in the patients with idiopathic nephrotic syndrome (4.6% higher than control, P = 0.719) by Al-Eisa and Haider [34]. However, literature is scarce regarding the association of TaqI CC genotype with the risk of hypertension in Bangladeshi population. For VDR BsmI genotypes, frequencies among the hypertensive patients were 9.91%, 57.66% and 32.43% for CC, CT and TT genotypes respectively (Table 3), whereas frequencies among control subjects were 10.0%, 58.0% and 32.0% respectively, suggesting no significant difference (p >0.05) of VDR BsmI genotypes between the studied subjects. The results of BsmI genotypes corroborate with previous results obtained from a study performed in Caucasian population with cardiovascular diseases [31]. Contrastively, studies by Jahromi et al. found a positive association of VDR BsmI genotypes with the risk of ischemic stroke, one type of cardiovascular diseases [35].

When analyzing VDR gene FokI genotype frequencies among study subjects, we found that hypertensive patients possess 9.65%, 34.21% and 56.14% of CC, CT and TT genotypes respectively. On the contrary, genotype frequencies of control subjects were found to be 9.0%, 56.0% and 35.0% respectively (Table 3). Interestingly, VDR gene FokI showed significant difference (p <0.01) between the hypertensive patients and healthy controls which is partly associated with several studies performed worldwide [31, 36].

Furthermore, we analyzed systolic blood pressure and diastolic blood pressure level in hypertensive patient and control group based on VDR genotypes. For both TaqI and BsmI genotypes, systolic blood pressure level in hypertensive patient group was significantly higher rather than control group (p <0.001). For two VDR FokI genotypes (CT and TT), systolic blood pressure level in hypertensive patient group was significantly higher rather than control subject (p <0.001). However, no significant difference between the two-study group (p >0.05) was found for FoKI CC genotype. Sequential analysis of diastolic blood pressure level depicted that DBP level was significantly higher in hypertensive patients concerning TaqI, BsmI and FokI (only CT and TT) genotypes. Statistical analysis of 25-hydroxy vitamin D level in different VDR genotypes suggest that 25-hydroxy vitamin D level in hypertensive patient was significantly lower rather than control subjects in all three VDR polymorphisms (p <0.001) (Table 4).

In addition, one-way ANOVA was used to analyze significant difference between different genotypic group in terms of biochemical parameters and baseline characteristics. No significant differences was found among TaqI, BsmI and FokI genotypic group in terms of Systolic Blood Pressure level (p >0.05). Similar results were obtained regarding diastolic blood pressure and BsmI as well as FokIi genotypes. Noteworthy that we found significant difference among VDR gene TaqI genotypic group in terms of Diastolic Blood Pressure level (p <0.05). No significant difference was found among 25-hydroxy vitamin D levels in all three VDR genotypes.

Finally, haplotype analysis revealed that TaqI and BsmI haplotypes showed no significant association with the risk of hypertension. For VDR gene TaqI and FokI, TC haplotype showed protective role in the risk of developing hypertension in patients (P<0.05, OR = 0.572, 95% CI = 0.361–0.907), whereas TT haplotype showed significantly 1.6 times increased risk of hypertension compared to controls (P<0.05, OR = 1.627, 95% CI = 1.053–2.515) (Table 5). It was hypothesized during the HapMap project that SNPs having high minor allele frequency are responsible for developing disease. Nevertheless, there are rare alleles that may also be responsible for disease. To see the combined effect of the alleles in disease development, haplotype analysis tells whether the SNPs are carried in the same haplotype block. We hope that our haplotype analysis of the VDR gene SNPs will identify SNP haplotypes whether they are carried under the same haplotype block and the impact of different haplogroups on disease risk.

Association of low vitamin D levels with an increased risk of hypertension is reported by many epidemiological studies. Renin Angiotensin Aldosterone System (RAAS) activation is the potential mechanism which contribute to the development of hypertension in vitamin D deficiency. Experimental evidence showed that damaging vitamin D receptors increase the activity of renin and circulating angiotensin II may lead to hypertension. This condition was reversed by blocking the RAAS [37, 38].

In conclusion, several studies have evaluated the association between VDR gene polymorphisms and the risk of hypertension, but the results remain convicting and inconsistent. In our study we found an association of TT genotype of VDR FokI between case and control and the risk of developing hypertension in Bangladeshi population (p <0.01) while we found no association with VDR gene TaqI and BsmI (P> 0.05). Furthermore, we observed decreased level of 25(OH)D in hypertensive patients suggesting that vitamin D deficiency may be one of the risk factors associated with hypertension in Bangladeshi population. Overall, the population-based case-control study suggests that there was statistically significant association between FokI polymorphism and risk of developing hypertension in Bangladeshi population. Further studies will be needed to warrant the potential use of vitamin D deficiency and VDR gene polymorphisms as a biomarker of hypertension.

## Supporting information

S1 File Supplementary figures SF1-SF4. (PPTX)

S2 File Supplementary tables ST1-ST8. (DOCX)

S3 File Uncropped original gel images SF5-SF7. (PPTX)
