# Supplementary material for: Vitamin D deficiency and the vitamin D receptor (VDR) gene polymorphism rs2228570 (FokI) are associated with an increased susceptibility to hypertension among the Bangladeshi population
Source: PLoS One. 2024 Mar 14;19(3):e0297138. doi: 10.1371/journal.pone.0297138 (PMC10939211; doi:10.1371/journal.pone.0297138)
Supplement: S1 File — (PPTX) [file pone.0297138.s001.pptx]

## Slide 1
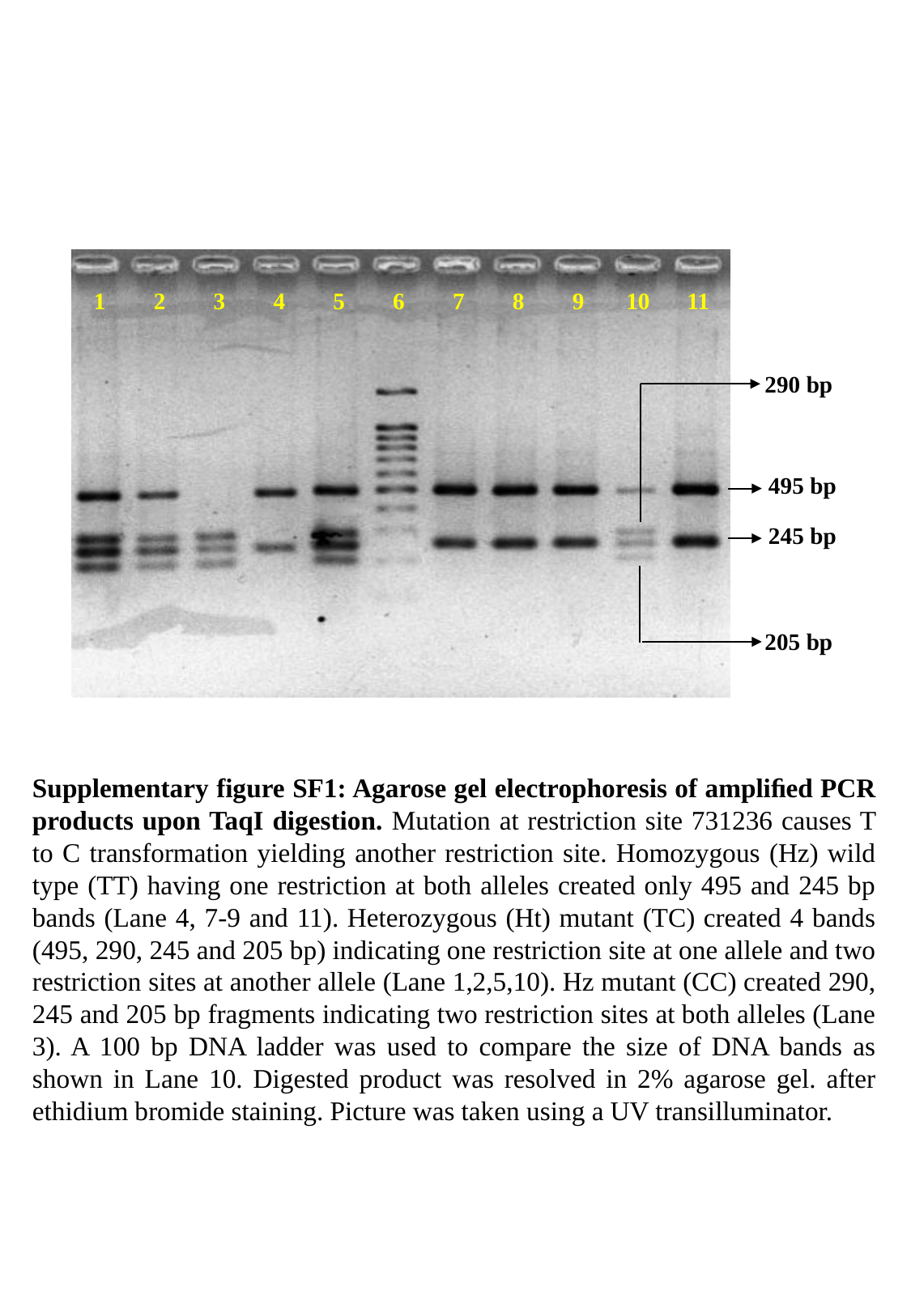

| 1 | 2 | 3 | 4 | 5 | 6 | 7 | 8 | 9 | 10 | 11 |
| --- | --- | --- | --- | --- | --- | --- | --- | --- | --- | --- |
290 bp
495 bp
245 bp
205 bp
Supplementary figure SF1: Agarose gel electrophoresis of ampliﬁed PCR products upon TaqI digestion. Mutation at restriction site 731236 causes T to C transformation yielding another restriction site. Homozygous (Hz) wild type (TT) having one restriction at both alleles created only 495 and 245 bp bands (Lane 4, 7-9 and 11). Heterozygous (Ht) mutant (TC) created 4 bands (495, 290, 245 and 205 bp) indicating one restriction site at one allele and two restriction sites at another allele (Lane 1,2,5,10). Hz mutant (CC) created 290, 245 and 205 bp fragments indicating two restriction sites at both alleles (Lane 3). A 100 bp DNA ladder was used to compare the size of DNA bands as shown in Lane 10. Digested product was resolved in 2% agarose gel. after ethidium bromide staining. Picture was taken using a UV transilluminator.

## Slide 2
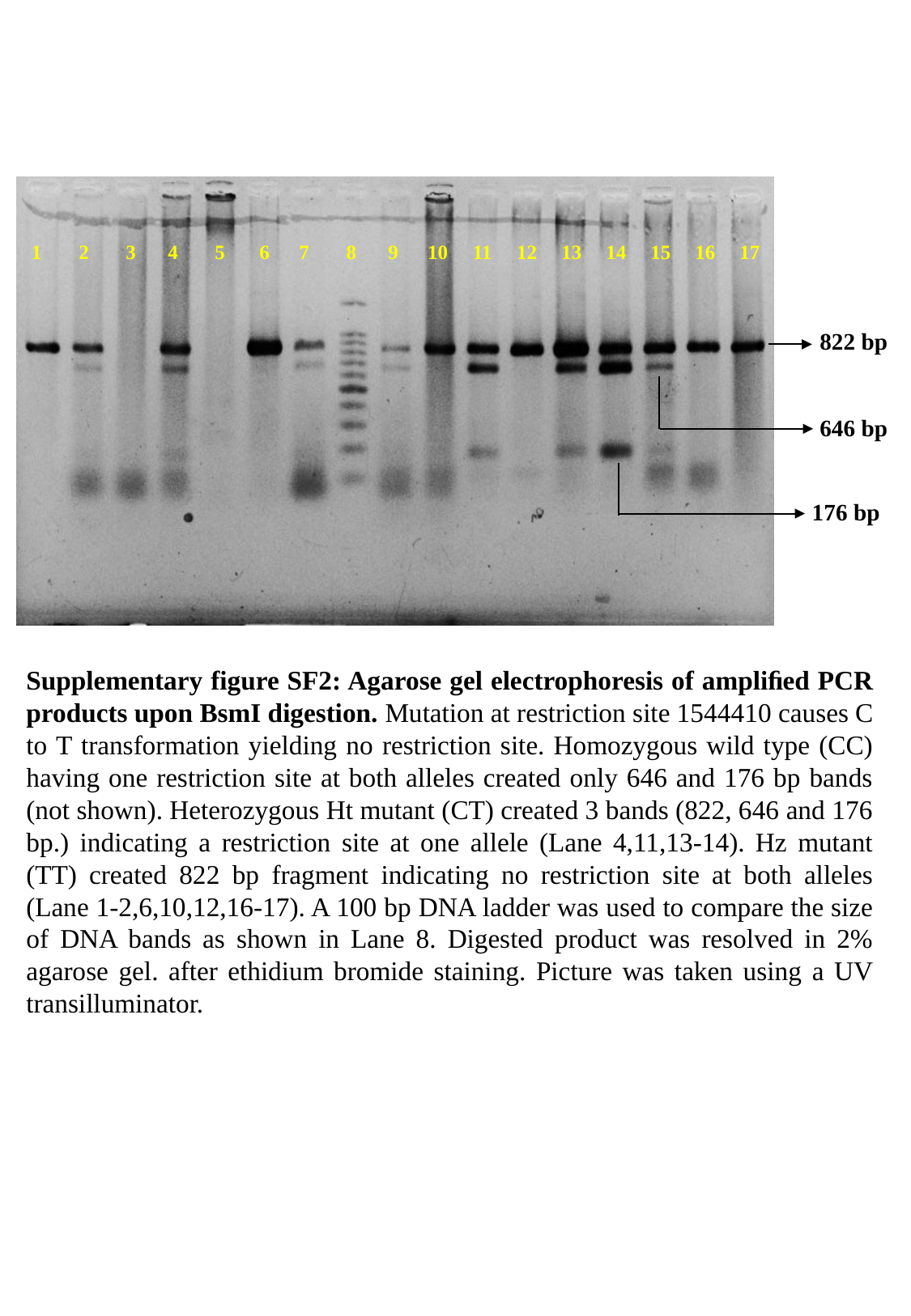

| 1 | 2 | 3 | 4 | 5 | 6 | 7 | 8 | 9 | 10 | 11 | 12 | 13 | 14 | 15 | 16 | 17 |
| --- | --- | --- | --- | --- | --- | --- | --- | --- | --- | --- | --- | --- | --- | --- | --- | --- |
822 bp
646 bp
176 bp
Supplementary figure SF2: Agarose gel electrophoresis of ampliﬁed PCR products upon BsmI digestion. Mutation at restriction site 1544410 causes C to T transformation yielding no restriction site. Homozygous wild type (CC) having one restriction site at both alleles created only 646 and 176 bp bands (not shown). Heterozygous Ht mutant (CT) created 3 bands (822, 646 and 176 bp.) indicating a restriction site at one allele (Lane 4,11,13-14). Hz mutant (TT) created 822 bp fragment indicating no restriction site at both alleles (Lane 1-2,6,10,12,16-17). A 100 bp DNA ladder was used to compare the size of DNA bands as shown in Lane 8. Digested product was resolved in 2% agarose gel. after ethidium bromide staining. Picture was taken using a UV transilluminator.

## Slide 3
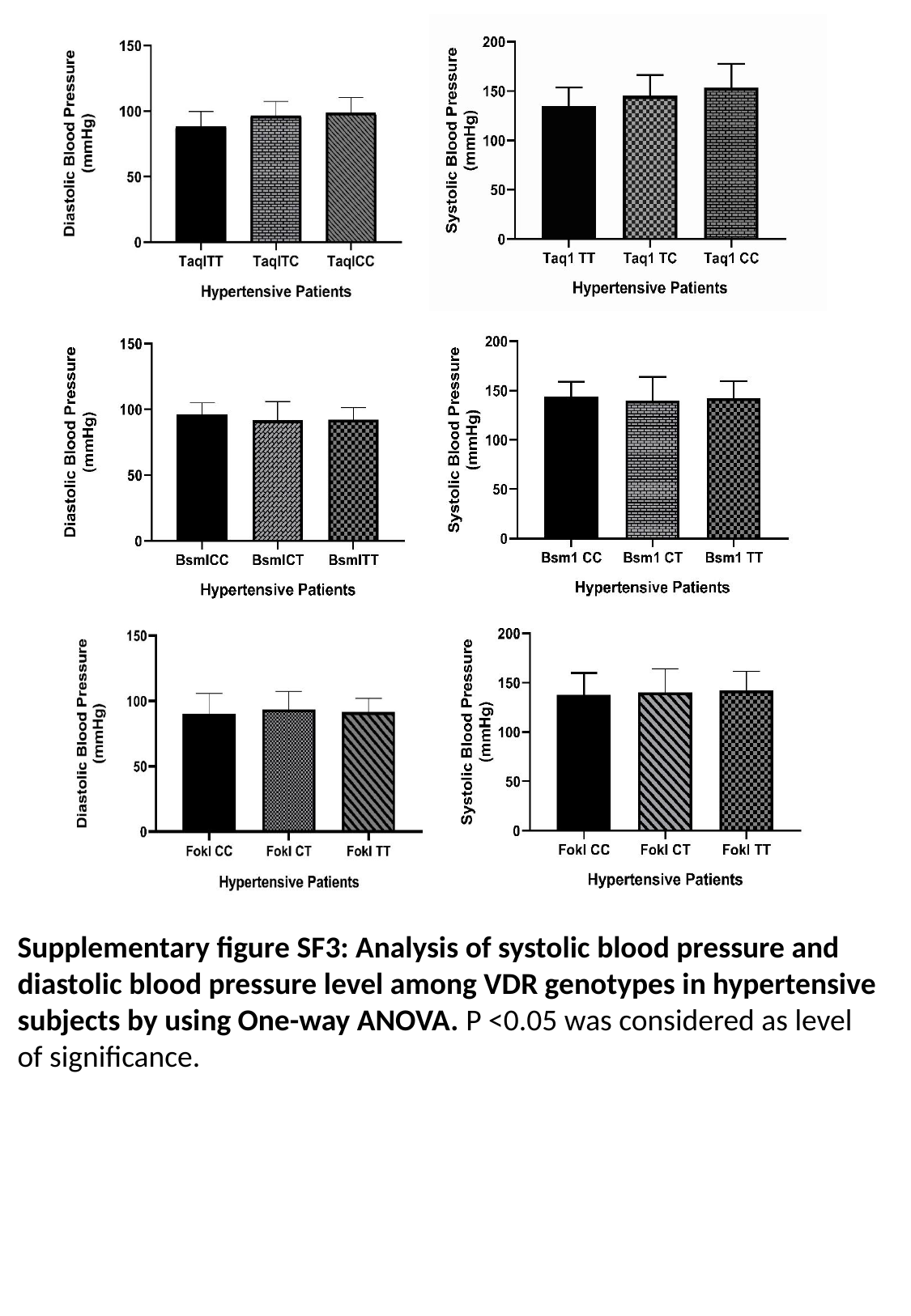

Supplementary figure SF3: Analysis of systolic blood pressure and diastolic blood pressure level among VDR genotypes in hypertensive subjects by using One-way ANOVA. P <0.05 was considered as level of signiﬁcance.

## Slide 4
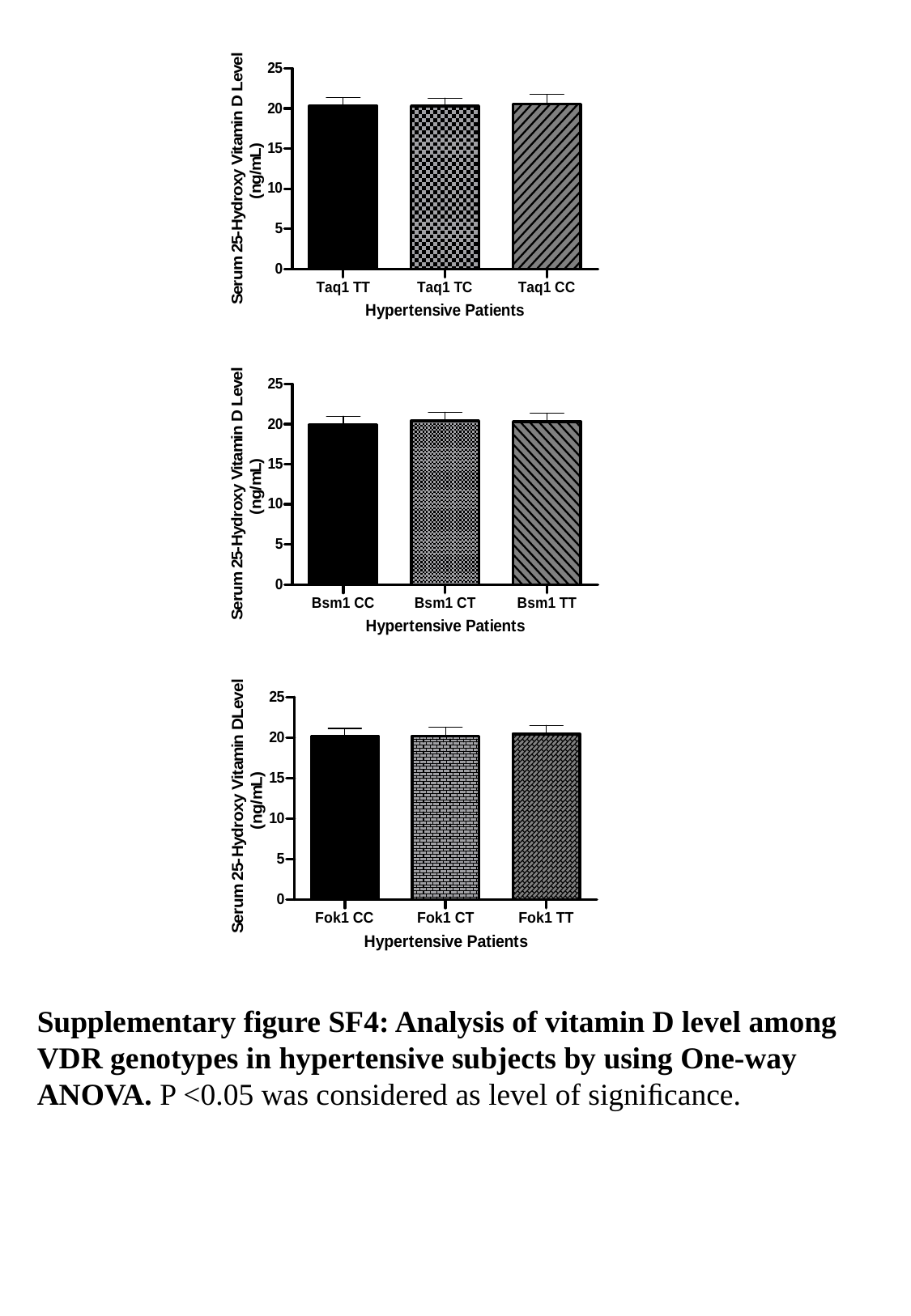

Supplementary figure SF4: Analysis of vitamin D level among VDR genotypes in hypertensive subjects by using One-way ANOVA. P <0.05 was considered as level of signiﬁcance.
