# Supplementary material for: Vitamin D deficiency and the vitamin D receptor (VDR) gene polymorphism rs2228570 (FokI) are associated with an increased susceptibility to hypertension among the Bangladeshi population
Source: PLoS One. 2024 Mar 14;19(3):e0297138. doi: 10.1371/journal.pone.0297138 (PMC10939211; doi:10.1371/journal.pone.0297138)
Supplement: S2 File — (DOCX) [file pone.0297138.s002.docx]

**Supplementary Table ST1:** Genotype frequency of VDR gene TaqI polymorphism and estimated risk with hypertension based on gender (For Male).

| **Genotype** | **Control**  **N (%)** | **Case**  **N (%)** | **P value^a^** | **P value^b^** | **OR (95% CI)** |
| --- | --- | --- | --- | --- | --- |
| TT | 30 (43.47) | 29 (38.16) | 0.3367 (ns) | - | 1 (Ref) |
| TC | 33 (47.83) | 34 (44.74) |  | >0.9999 (ns) | 1.075 (0.5020-2.296) |
| CC | 6 (8.69) | 13 (17.11) |  | 0.2486 (ns) | 2.466 (0.7259-7.589) |
| TC+CC  (Dominant Model) | 39 (56.52) | 47 (61.84) |  | 0.5794 (ns) | - 1. .6361-2.573) |

Data represented as number (percentage), N (%). Chi-square and fisher’s exact test were done to assess significant difference between genotypic group and association of genotype with disease condition. OR (95% CI); Odds Ratio (95% Confidence Interval). P value^a^ for chi-square test. P value^b^ for fisher’s exact test.

**Supplementary Table ST2:** Genotype frequency of VDR gene TaqI polymorphism and estimated risk with hypertension based on gender (For Female).

| **Genotype** | **Control**  **N (%)** | **Case**  **N (%)** | **P value^a^** | **P value^b^** | **OR (95% CI)** |
| --- | --- | --- | --- | --- | --- |
| TT | 13 (41.94) | 22 (62.86) | 0.2020 (ns) | **-** | 1 (Ref) |
| TC | 16 (51.61) | 10 (28.57) |  | 0.1458 (ns) | 0.3719 (0.1248-1.217) |
| CC | 2 (6.45) | 3 (8.57) |  | **>**0.9999 (ns) | 1.227 (0.1624-17.53) |
| TC+CC  (Dominant Model) | 18 (58.06) | 13 (37.14) |  | 0.1733 (ns) | 0.4432 (0.1565-1.308) |

Data represented as number (percentage), N (%). Chi-square and fisher’s exact test were done to assess significant difference between genotypic group and association of genotype with disease condition. OR (95% CI); Odds Ratio (95% Confidence Interval). P value^a^ for chi-square test. P value^b^ for fisher’s exact test.

**Supplementary Table ST3:** Genotype frequency of VDR gene BsmI polymorphism and estimated risk with hypertension based on gender (For Male).

| **Genotype** | **Control**  **(N%)** | **Case**  **(N%)** | **P-value^a^** | **P-value^b^** | **OR (95% CI)** |
| --- | --- | --- | --- | --- | --- |
| CC | 7 (10.14) | 7 (9.33) | 0.7996 (ns) | - | 1 (Ref) |
| CT | 34 (49.28) | 42 (56.0) |  | 0.7587 (ns) | 1.200 (0.3762-4.074) |
| TT | 28 (40.58) | 26 (34.67) |  | >0.9999 (ns) | 0.9286 (0.2747-3.399) |
| CT+TT  (Dominant Model) | 62 (89.86) | 68 (90.67) |  | >0.9999 (ns) | 1.079 (0.3619-3.384) |

Data represented as number (percentage), N (%). Chi-square and fisher’s exact test were done to assess significant difference between genotypic group and association of genotype with disease condition. OR (95% CI); Odds Ratio (95% Confidence Interval). P value^a^ for chi-square test. P value^b^ for fisher’s exact test**.**

**Supplementary Table ST4:** Genotype frequency of VDR gene BsmI polymorphism and estimated risk with hypertension based on gender (For Female).

| **Genotype** | **Control**  **(N%)** | **Case**  **(N%)** | **P-value^a^** | **P-value^b^** | **OR (95% CI)** |
| --- | --- | --- | --- | --- | --- |
| CC | 3 (9.68) | 4 (11.11) | 0.4624 (ns) | - | 1 (Ref) |
| CT | 24 (77.42) | 22 (61.11) |  | >0.9999 (ns) | 0.6875 (0.1204-3.332) |
| TT | 4 (12.9) | 10 (27.78) |  | >0.9999 (ns) | 1.667 (0.2222-10.71) |
| CT+TT | 28 (90.32) | 32 (88.89) |  | >0.9999 (ns) | 0.8421 (0.1496-3.920) |

Data represented as number (percentage), N (%). Chi-square and fisher’s exact test were done to assess significant difference between genotypic group and association of genotype with disease condition. OR (95% CI); Odds Ratio (95% Confidence Interval). P value^a^ for chi-square test. P value^b^ for fisher’s exact test.

**Supplementary Table ST5:** Genotype frequency of VDR gene FokI polymorphism and estimated risk with hypertension based on gender (For Male).

| **Genotype** | **Control**  **(N%)** | **Case**  **(N%)** | **P-value^a^** | **P-value^b^** | **OR (95% CI)** |
| --- | --- | --- | --- | --- | --- |
| CC | 8 (11.59) | 5 (6.58) | 0.0379 (P<0.05) | - | 1 (Ref) |
| CT | 37 (53.62) | 27 (35.53) |  | >0.9999 (ns) | 1.117 (0.3222-3.791) |
| TT | 24 (34.78) | 44 (57.89) |  | 0.1667 (ns) | 2.779 (0.7837-9.460) |
| CT+TT  (Dominant Model) | 61 (88.4) | 71 (93.42) |  | 0.5242 (ns) | 1.775 (0.4785-5.489) |

Data represented as number (percentage), N (%). Chi-square and fisher’s exact test were done to assess significant difference between genotypic group and association of genotype with disease condition. OR (95% CI); Odds Ratio (95% Confidence Interval). P value^a^ for chi-square test. P value^b^ for fisher’s exact test.

**Supplementary Table ST6:** Genotype frequency of VDR gene FokI polymorphism and estimated risk with hypertension based on gender (For Female).

| **Genotype** | **Control**  **(N%)** | **Case**  **(N%)** | **P-value^a^** | **P-value^b^** | **OR (95% CI)** |
| --- | --- | --- | --- | --- | --- |
| CC | 2 (6.45) | 6 (15.79) | 0.0665 (ns) | - | 1 (Ref) |
| CT | 19 (61.29) | 12 (31.58) |  | 0.1037 (ns) | 0.1538 (0.0126-1.369) |
| TT | 10 (32.26) | 20 (52.63) |  | >0.9999 (ns) | 0.4762 (0.0368-3.561) |
| CT+TT | 29 (93.55) | 32 (84.21) |  | 0.4032 (ns) | 0.2667 (0.0223-1.949) |

Data represented as number (percentage), N (%). Chi-square and fisher’s exact test were done to assess significant difference between genotypic group and association of genotype with disease condition. OR (95% CI); Odds Ratio (95% Confidence Interval). P value^a^ for chi-square test. P value^b^ for fisher’s exact test.

**Supplementary table ST7: Genotypic distribution of systolic blood pressure level in control and hypertensive patient group.**

| **Genotypes** | **Systolic Blood Pressure (mmHg)** | | **P-value** |
| --- | --- | --- | --- |
|  | **Case** | **Control** |  |
| **TaqI VDR Gene** | | | |
| **TT** | 134.8 ± 3.596 | 109.1 ± 3.149 | P <0.001 |
| **TC** | 145 ± 4.382 | 112.3 ± 2.571 | P <0.001 |
| **CC** | 153.3 ± 9.888 | 120.0 ± 3.651 | P <0.05 |
| **BsmI VDR Gene** | | | |
| **CC** | 144.0 ± 6.782 | 110.0 ± 7.071 | P <0.05 |
| **CT** | 139.9 ± 4.129 | 112.0 ± 2.430 | P <0.001 |
| **TT** | 142.1 ± 4.016 | 110 ± 2.673 | P <0.001 |
| **FokI VDR Gene** | | | |
| **CC** | 138.0 ± 9.695 | 113.3 ± 8.819 | ns |
| **CT** | 140.0 ± 4.890 | 110.0 ± 2.760 | P <0.001 |
| **TT** | 142.2 ± 3.592 | 111.4 ± 1.429 | P <0.001 |

Data represented as mean ± SEM. Unpaired t-test was used to determine significant difference between the two groups. p <0.05 taken as level of significance. ns= non-significant (P >0.05)

**Supplementary table ST8: Genotypic distribution of diastolic blood pressure level in control and hypertensive patient group.**

| **Genotypes** | **Diastolic Blood Pressure (mmHg)** | | **P-value** |
| --- | --- | --- | --- |
|  | **Case** | **Control** |  |
| **TaqI VDR Gene** | | | |
| **TT** | 87.86 ± 2.248 | 73.64 ± 2.344 | P <0.001 |
| **TC** | 96.04 ± 2.292 | 75.77 ± 2.254 | P <0.001 |
| **CC** | 98.33 ± 4.773 | 78.75 ± 3.146 | P <0.05 |
| **BsmI VDR Gene** | | | |
| **CC** | 96.0 ± 4.0 | 72.50 ± 4.330 | P <0.01 |
| **CT** | 91.76 ± 2.433 | 73.33 ± 2.108 | P <0.001 |
| **TT** | 92.37 ± 2.072 | 79.29 ± 1.700 | P <0.01 |
| **FokI VDR Gene** | | | |
| **CC** | 90.0 ± 7.071 | 75.0 ± 6.455 | ns |
| **CT** | 93.54 ± 2.831 | 74.33 ± 1.944 | P <0.001 |
| **TT** | 91.72 ± 1.927 | 76.25 ± 1.830 | P <0.001 |
|  |  |  |  |

Data represented as mean ± SEM. Unpaired t-test was used to determine significant difference between the two groups. p <0.05 taken as level of significance**.** ns= non-significant (P >0.05)
