# Supplementary material for: Vitamin D deficiency and the vitamin D receptor (VDR) gene polymorphism rs2228570 (FokI) are associated with an increased susceptibility to hypertension among the Bangladeshi population
Source: PLoS One. 2024 Mar 14;19(3):e0297138. doi: 10.1371/journal.pone.0297138 (PMC10939211; doi:10.1371/journal.pone.0297138)
Supplement: S3 File — (PPTX) [file pone.0297138.s003.pptx]

## Slide 1
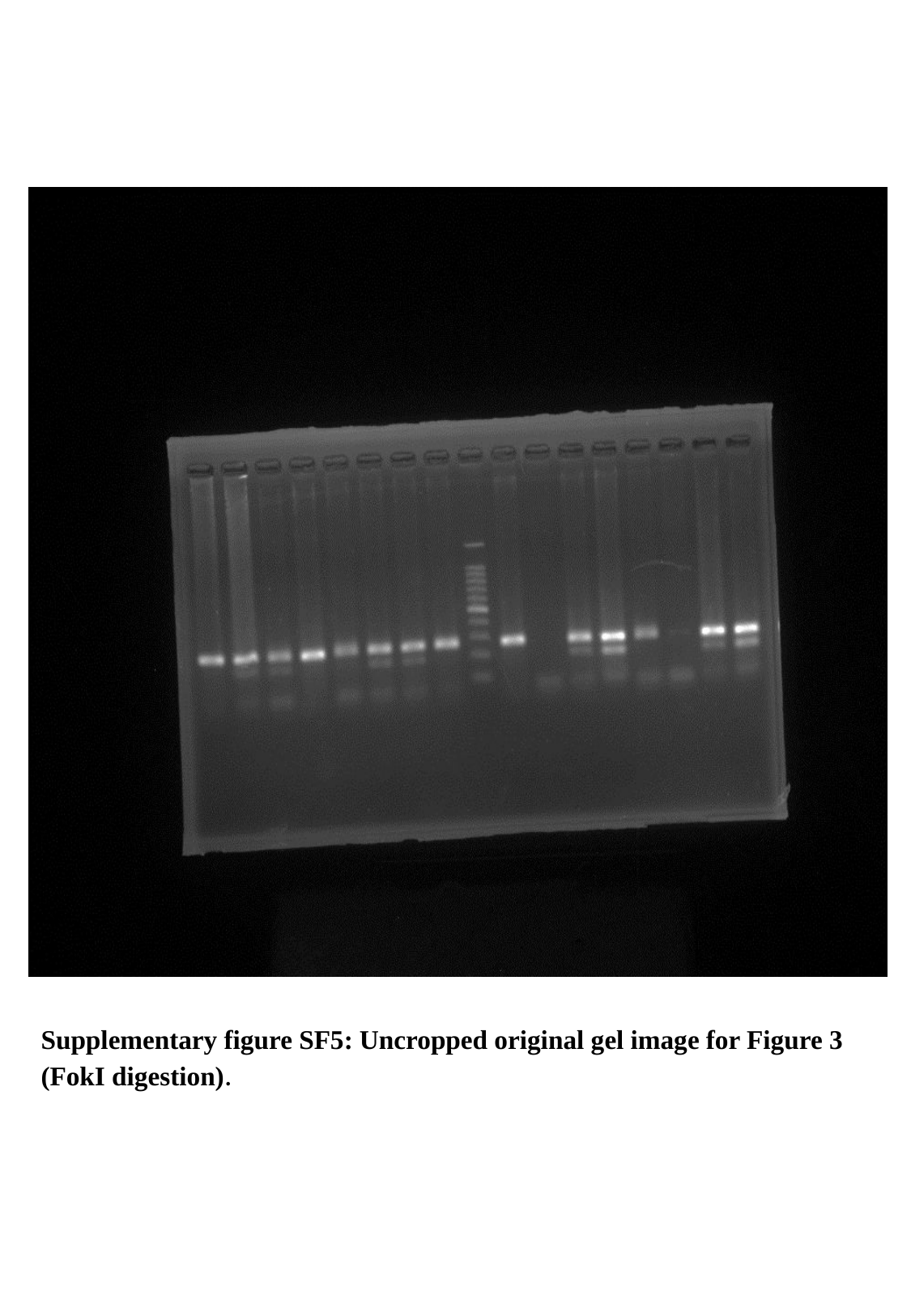

Supplementary figure SF5: Uncropped original gel image for Figure 3
(FokI digestion).

## Slide 2
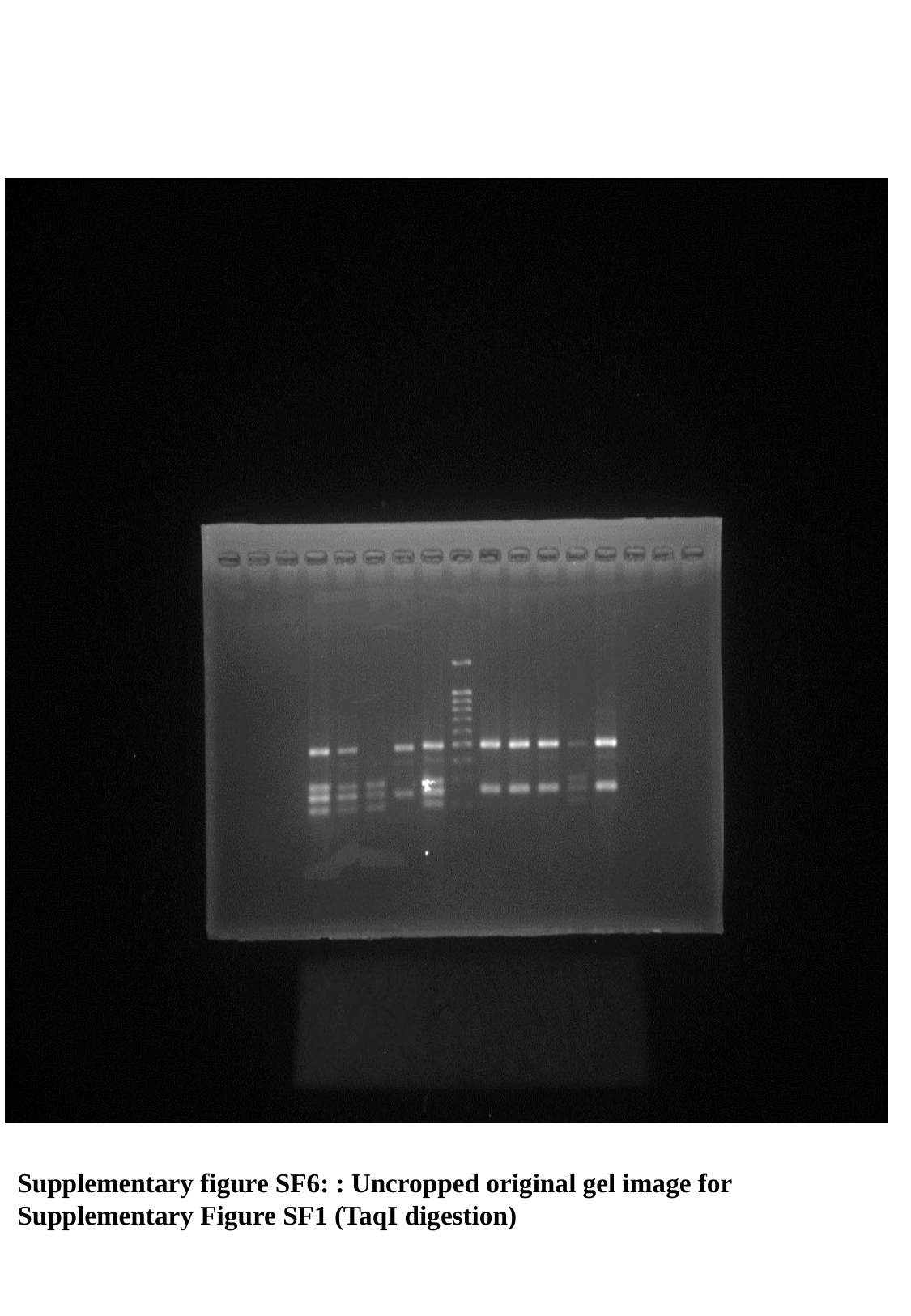

Supplementary figure SF6: : Uncropped original gel image for Supplementary Figure SF1 (TaqI digestion)

## Slide 3
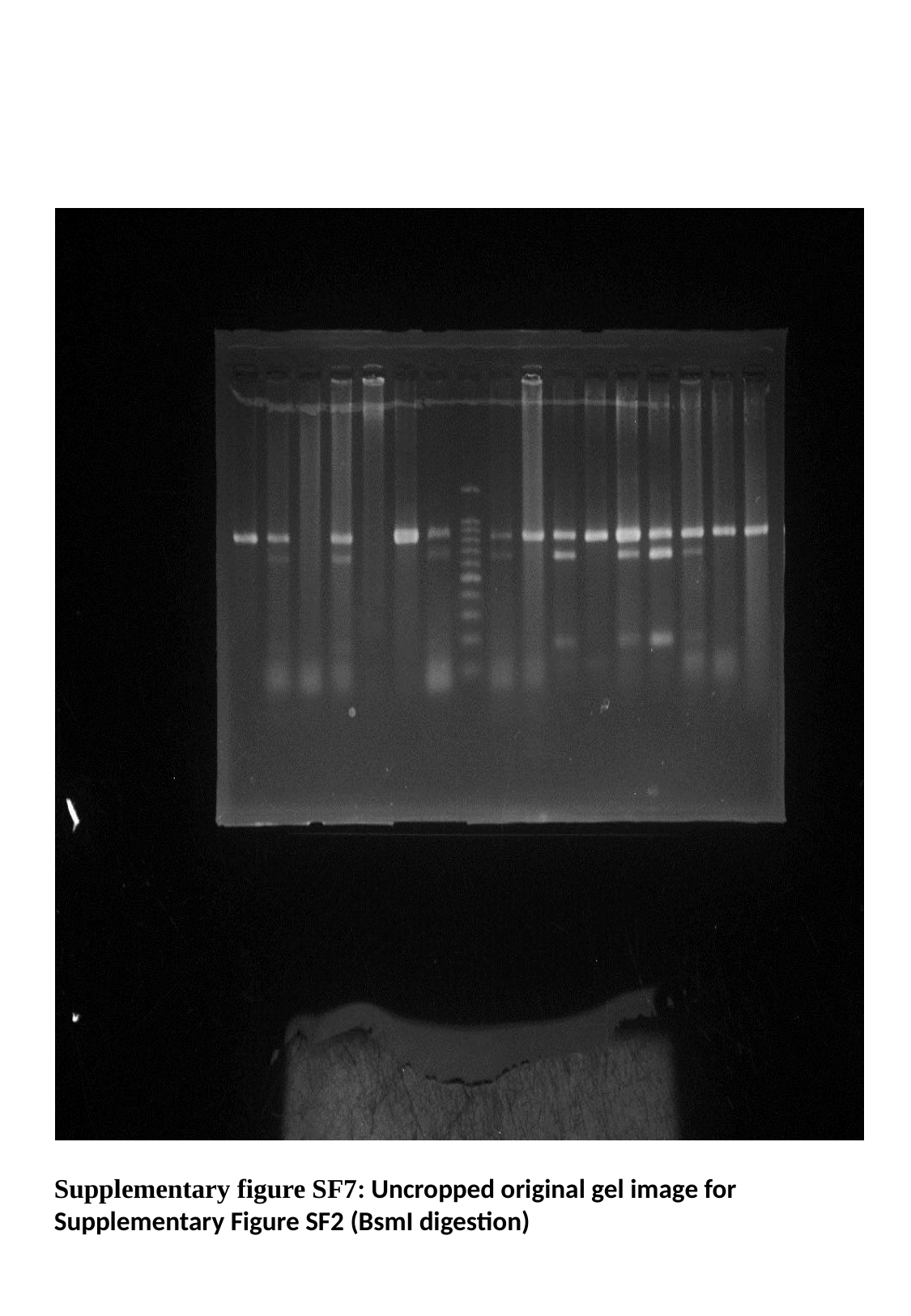

Supplementary figure SF7: Uncropped original gel image for Supplementary Figure SF2 (BsmI digestion)
